# Nitric Acid in Whooping-Cough

**Published:** 1873-06

**Authors:** 


Nitric Acid in Whooping-Cough.—This self-limited, but generally incurable disease, is sometimes treated by the empirical use of nitric acid. We say empirical because the remedy is given by the advice of others, without the most remote idea of its mode of action. Dr. Berry, of Lancaster, as we find in the *Reporter*, Philadelphia, considers it a "safe, simple, and apparently valuable" remedy in the treatment of complicated whooping-cough. Whether the relief was in consequence of the remedy, he was not sure—only knows that a cure followed its administration, and has no definite idea of the *modus operandi*.

He believes the acid to be a "tonic, [to the digestive organs, we suppose,] sedative, [to heart or nervous centers?] and antiseptic." He would not have its refrigerative effects lost sight of. Now, it is known that nitric acid is a gastric and hepatic or general digestive tonic. Is it by virtue of this property that whooping-cough is cured, or by some other action of the remedy not very well established? What is the pathology of the disease? Is the primary disturbance in the air passages?

We have reason to believe the remedy acts favorably to the relief of this troublesome malady, in the dose of one drop or less, three times a day, for an adult, but whether the impression is made directly upon the respiratory apparatus, or some other part, in affording this therapeutical result is yet to be determined.

